# Bisphenol S Impairs Invasion and Proliferation of Extravillous Trophoblasts Cells by Interfering with Epidermal Growth Factor Receptor Signaling

**DOI:** 10.3390/ijms23020671

**Published:** 2022-01-08

**Authors:** Elvis Ticiani, Yong Pu, Jeremy Gingrich, Almudena Veiga-Lopez

**Affiliations:** 1 Department of Pathology, University of Illinois at Chicago, Chicago, IL 60612, USA; eticiani@uic.edu (E.T.); yongpu@uic.edu (Y.P.); 2 Department of Pharmacology and Toxicology, Michigan State University, East Lansing, MI 48824, USA; gingric9@msu.edu; 3 The Chicago Center for Health and the Environment, University of Illinois at Chicago, Chicago, IL 60612, USA

**Keywords:** bisphenol S, placenta, extravillous trophoblasts, membrane receptor

## Abstract

The placenta supports fetal growth and is vulnerable to exogenous chemical exposures. We have previously demonstrated that exposure to the emerging chemical bisphenol S (BPS) can alter placental endocrine function. Mechanistically, we have demonstrated that BPS interferes with epidermal growth factor receptor (EGFR) signaling, reducing placenta cell fusion. Extravillous trophoblasts (EVTs), a placenta cell type that aids with vascular remodeling, require EGF to invade into the maternal endometrium. We hypothesized that BPS would impair EGF-mediated invasion and proliferation in EVTs. Using human EVTs (HTR-8/SVneo cells), we tested whether BPS could inhibit the EGF response by blocking EGFR activation. We also evaluated functional endpoints of EGFR signaling, including EGF endocytosis, cell invasion and proliferation, and endovascular differentiation. We demonstrated that BPS blocked EGF-induced phosphorylation of EGFR by acting as a competitive antagonist to EGFR. Transwell assay and a three-dimensional microfluidic chip invasion assay revealed that BPS exposure can block EGF-mediated cell invasion. BPS also blocked EGF-mediated proliferation and endovascular differentiation. In conclusion, BPS can prevent EGF-mediated EVT proliferation and invasion through EGFR antagonism. Given the role of EGFR in trophoblast proliferation and differentiation during placental development, our findings suggest that maternal exposure to BPS may contribute to placental dysfunction via EGFR-mediated mechanisms.

## 1. Introduction

Epidemiological studies increasingly support the link between gestational exposure to endocrine-disrupting chemicals (EDCs) and placental dysfunction [1,2]. EDCs are of concern because they can interfere with endogenous hormone signaling [3]. Among these EDCs are bisphenols; compounds that are used in the production of plastics and manufacturing of consumer products [4]. Bisphenols are high-production-volume chemicals [5]. Bisphenol A (BPA) and bisphenol S (4,4′-sulfonyldiphenol; BPS) are the most abundant bisphenol congeners detected in humans, with BPS being present in over 80% of human urine samples from the United States [6] and six other Asian countries [6,7,8,9], ranging from 0.07 to 211.9 ng/mL [10]. BPS is also present in foodstuffs [11], indoor dust [12], sewage sludge [13], ground water [14], and sediments from industrialized areas [8]. Growing evidence supports the notion that BPS exposure can affect human health [15,16,17]. Additionally, previous studies have demonstrated that BPS can increase estrogen-responsive gene expression in the ovary and uterus, interfering with the development of the reproductive tract [18]. Particularly concerning are exposures that occur during pregnancy, since bisphenol chemicals can not only reach and target the developing fetus [19], but also the placenta [2]. BPA [20,21,22] and, more recently, BPS have been reported to affect placental morphology, cell fusion, and nutrient transport [16,22,23,24].

Though transient in nature, the placenta is essential for pregnancy maintenance [25]. Comprised of multiple cell types, such as, cytotrophoblasts, macrophages, stromal, and endometrial cells, the placenta orchestrates fetal growth through regulation of hormone synthesis, immune function, and nutrient and oxygen transport between the mother and the fetus [26]. Cytotrophoblasts are cells that populate the villous tree at the feto–maternal interface and differentiate into two distinct cell populations: syncytiotrophoblasts and extravillous trophoblasts (EVTs). Syncytiotrophoblasts are multinucleated cells that comprise the placental layer responsible for nutrient transfer across the feto–maternal interface. EVTs on the other hand, connect the placenta to the maternal endometrium by growing away from the villi in columns and invading into the maternal decidua [27]. EVTs connect the placenta to the maternal decidua and help transform the maternal spiral arteries into conduits capable of providing an adequate blood supply to the placenta and fetus [28]. Dysregulation of the EVT invasion process can result in a wide spectrum of pregnancy complications such as placenta accreta, increta, and percreta, as well as preeclampsia [29,30]. Thus, proper EVT invasion is paramount for maternal health and adequate development of the fetus.

Epidermal growth factor receptor (EGFR) signaling is required for proper control of cytotrophoblast growth and differentiation during placental development [31,32]. Aside from tumorigenic tissues, the placenta is one of the tissues with the highest abundance of EGFR [33]. This highlights EGFR’s key role in numerous placental cell processes, such as proliferation, fusion, and invasion [34,35]. EGFR, a transmembrane receptor, is activated by its endogenous ligand EGF, resulting in phosphorylation of EGFR and of the enzymes MAPK and JAK/STAT [36]. Activation of these kinases mediate gene transcription of matrix metalloproteinases (MMP) -2 and -9, stimulating motility and invasion of EVTs [37]. Chemicals such as BPA and methoxychlor can induce EGFR phosphorylation [38], while others, such as atrazine and polychlorinated biphenyls (e.g.,: PCB-153) can block it [39]. Recently, we have demonstrated that the emerging bisphenol, BPS, competitively binds to EGFR [24]. BPS also blocks EGFR signaling, fully attenuating EGF-mediated fusion in primary human cytotrophoblast cells [24]. Given the role of EGFR in stimulating EVT proliferation and invasion into the maternal decidua to promote endometrial vasculature remodeling [40], we hypothesized that BPS could interfere in EGF-mediated EVT invasion and proliferation. To test this, we used a combination of approaches, including quantification of protein expression and EGF endocytosis, as well as functional assays such as cell proliferation, tube forming, and invasion, which was evaluated using the classic Transwell assay and a state-of-the-art three-dimensional microfluidic platform in a first-trimester EVT cell line.

## 2. Results

### 2.1. BPS Inhibits EGFR Phosphorylation

**Figure 1:**
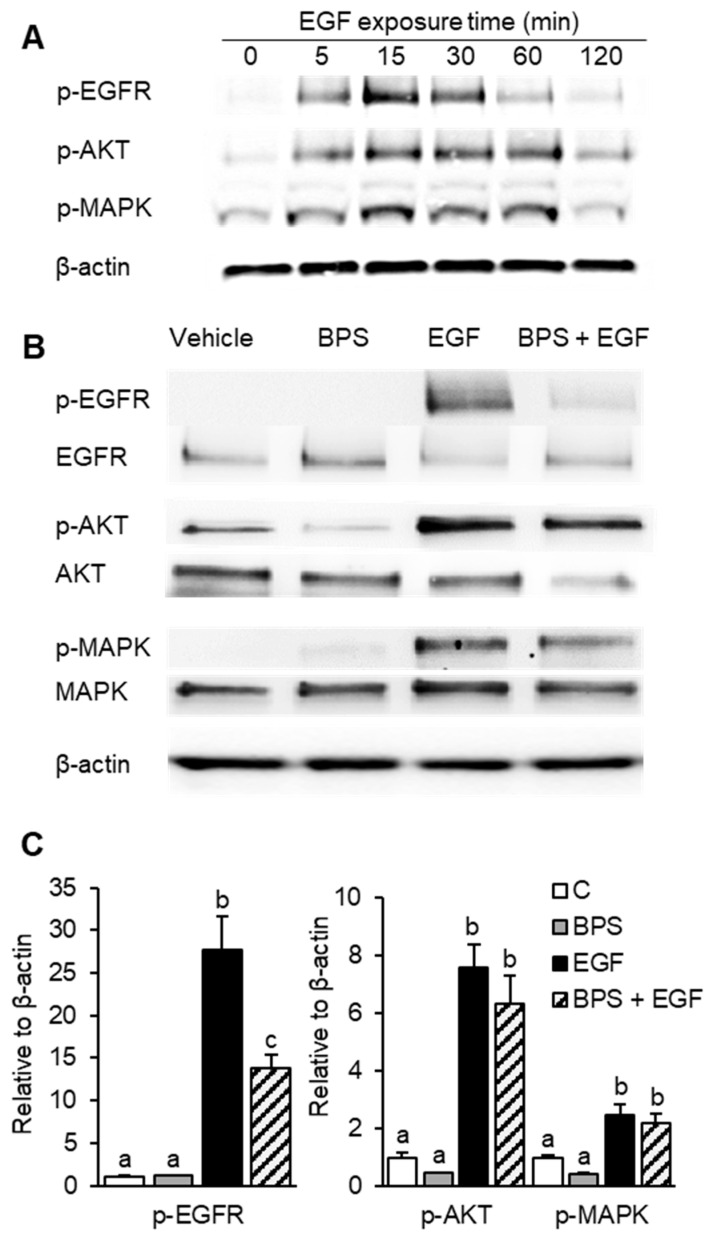
Effect of BPS exposure on EGF-mediated EGFR pathway activation in HTR-8/SVneo cells. (**A**) Representative Western blot images for total and phosphorylated proteins and the reference protein β-actin upon exposure to 30 ng/mL of EGF over time. (**B**) Representative Western blot images and (**C**) quantification (mean ± SEM) of total and phosphorylated EGFR, AKT and MAPK, and β-actin upon exposure to 0.1% DMSO (control; C), BPS (1000 ng/mL; graybars), EGF (30 ng/mL; closed bars), or BPS + EGF (stripped bars) treatments. A generalized linear model was used to statistically compare treatments. Different letters denote statistical differences among treatment groups at *p* < 0.05 (*n* = 3 technical replicates/group). BPS: bisphenol S, EGF: epidermal growth factor, p-EGFR: phospho-EGF receptor, p-AKT: phospho-protein kinase B, and p-ERK: phospho-extracellular receptor kinase.

We first determined that after exposure of HTR-8/SVneo cells for 0, 5, 15, 30, 60, or 120 min, the highest EGFR phosphorylation occurred after 15 min (Figure 1A). Thus, we used the 15 min exposure time to investigate if BPS’ ability to block EGF-mediated signaling occurred through a direct interaction with EGFR. The exposure of HTR-8/SVneo cells to EGF for 15 min led to an ~27-fold upregulation of p-EGFR compared to the control group (*p* < 0.01, Figure 1B,C). No effect was observed when cells were exposed to BPS alone, but co-exposure to EGF + BPS reduced p-EGFR by 2-fold compared to the EGF group (*p* < 0.01). Similar effects were observed in EGFR downstream effectors, where EGF upregulated p-AKT and p-MAPK by 7- and 3-fold, respectively. However, BPS was not able to attenuate AKT and MAPK phosphorylation when HTR-8/SVneo cells were co-incubated with EGF + BPS.

### 2.2. BPS Reduces EGF Internalization

**Figure 2:**
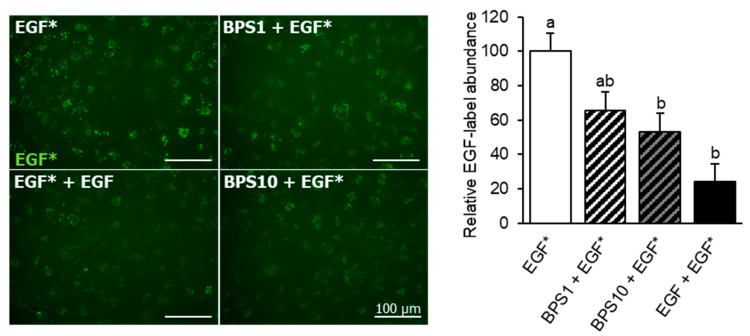
Effect of BPS on EGF internalization in HTR-8/SVneo cells. Representative images (**left**) of EGF in HTR-8/SVneo cells, and quantification (mean ± SEM) (**right**) following exposure to: (1) EGF* (100 ng/mL Alexa Fluor 647-labelled EGF), (2) BPS1 + EGF* (1 µg/mL BPS + 100 ng/mL EGF*), (3) BPS10 + EGF* (10 µg/mL BPS + 100 ng/mL EGF*) and (4) EGF + EGF* (100 ng/mL non-labelled EGF + 100 ng/mL EGF*). Images were taken 1 h after a 5 min exposure. Results were normalized by the total number of cells, and expressed as relative percentage of the EGF* group. A generalized linear model was used to statistically compare treatments. Different letters denote statistical differences among treatment groups at *p* < 0.05 (*n* = 4 technical replicates/group).

To determine if BPS acts as competitive EGFR antagonist, we evaluated its ability to displace bound fluorescent EGF from the EGFR binding site through a competitive EGF internalization assay. HTR-8/SVneo cells decreased internalization of Texas Red-tagged EGF in a dose-depended manner, after exposure to 1 or 10 μg/mL BPS (Figure 2). The reduction in EGF internalization in HTR-8/SVneo cells was 26% (*p* < 0.05) and 41% (*p* < 0.01) after exposure to 1 and 10 μg/mL BPS, respectively.

### 2.3. BPS Impairs EGF-Mediated Cell Proliferation

**Figure 3:**
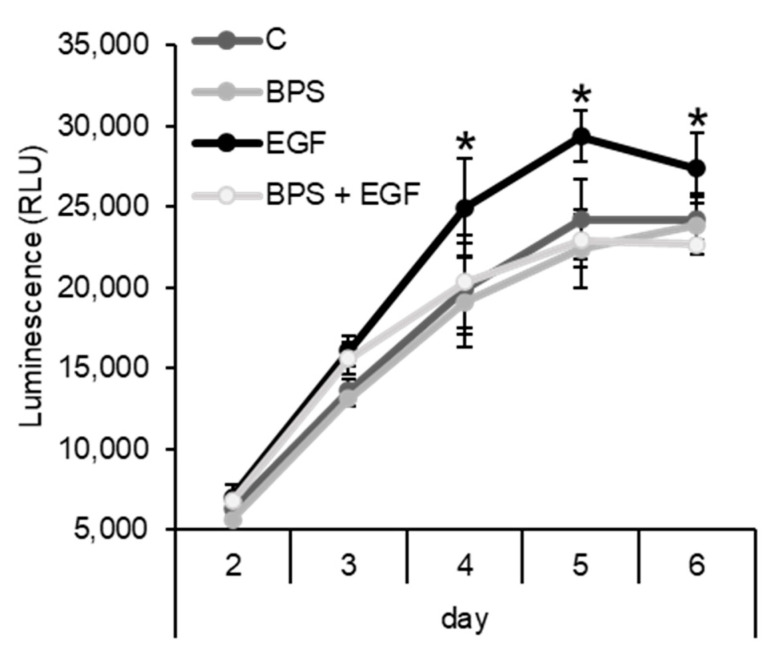
Effect of BPS on HTR-8/SVneo cell proliferation using CellTiter-Glo luminescent cell proliferation assay. Quantification expressed as relative light units (RLU) of HTR-8/SVneo cells exposed to 0.1% DMSO (control; C), BPS (1000 ng/mL), EGF (30 ng/mL;), or BPS + EGF (mean ± SEM) treatments. Generalized linear model was used compare treatments. Asterisks denotes statistical differences to the control group at *p* < 0.05 (*n* = 4 technical replicates/group).

To test whether BPS can affect HTR-8/SVneo cell’s ability to proliferate, or interfere with EGF-mediated proliferation, cells were exposed to BPS in the presence or absence of recombinant human EGF. EGF increased HTR-8/SVneo cell’s proliferation compared to the control group (*p* < 0.05, Figure 3) after 4 days in culture. BPS exposure for 2 to 6 days did not change the proliferation rate compared to the control group. However, BPS blocked EGF-mediated proliferation (*p* < 0.05) to the level of the control and BPS-only groups.

### 2.4. BPS Impairs EGF-Mediated Cell Invasion

**Figure 4:**
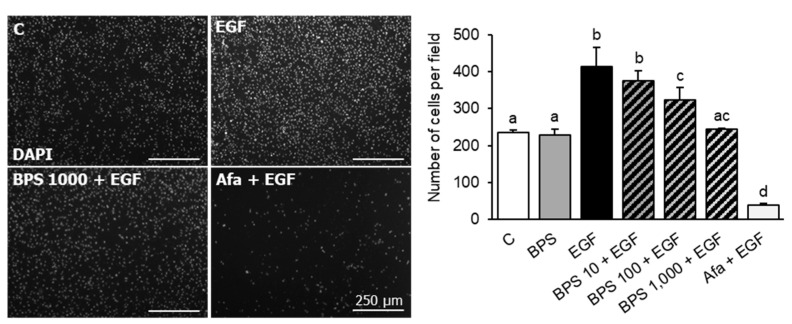
Effect of BPS on HTR-8/SVneo cell invasion using Matrigel pre-coated Transwell cell culture inserts. Representative images (**left**) of HTR-8/SVneo invasive cells, and quantification (mean ± SEM) (**right**) following exposure to 0.1% DMSO (control; C), BPS (1000 ng/mL), EGF (30 ng/mL), BPS (10, 100, or 1000 ng/mL) + EGF, or afatinib (Afa, 100 ng/mL) + EGF treatments. Cell invasion (mean ± SEM) was expressed as number of cells counted per field. A generalized linear model was used to compare treatments. Different letters denote statistical differences among treatment groups at *p* < 0.05 (*n* = 4 technical replicates/group).

**Figure 5:**
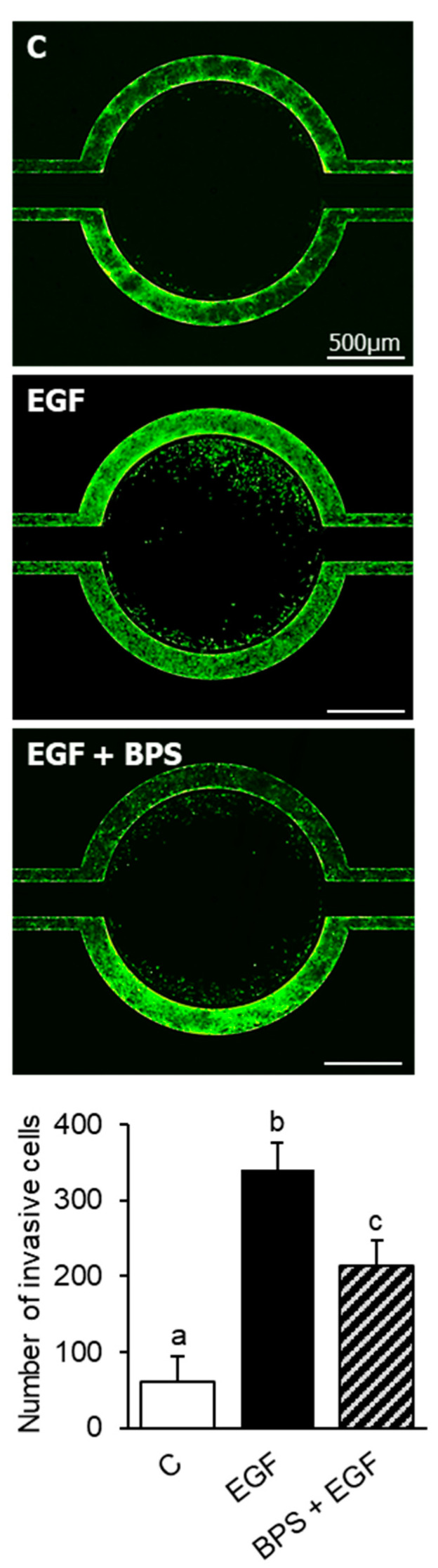
Effect of BPS on HTR-8/SVneo cell invasion using a 3D PDMS microfluidic chip. Representative images (**top**) of invasive HTR-8/SVneo GFP tagged cells, and quantification (mean ± SEM) (**bottom**) following exposure to 0.1% DMSO (control; C), EGF (30 ng/mL), or BPS (1000 ng/mL) + EGF treatments. Quantification (mean ± SEM) is expressed as number of invasive cells. A generalized linear model was used to compare treatments. Different letters denote statistical differences among treatment groups at *p* < 0.05 (*n* = 4 technical replicates/group).

To investigate BPS’ ability to block EGF-mediated cell invasion in EVTs, HTR-8/SVneo cells were exposed to BPS at increasing doses in combination with the EGFR antagonist, afatinib, in a Transwell cell culture system. EGF induced (*p* < 0.01) and afatinib blocked cell invasion compared to the control group (*p* < 0.01). BPS exposure blocked EGF-mediated cell invasion in a dose-dependent manner, reducing cell invasion to the level of the control at the 1000 ng/mL dose (Figure 4). To further confirm BPS’ ability to block EGF-mediated cell invasion, we tested BPS in a recently developed 3D microfluidic platform with constant media flow. The invasiveness of HTR-8/SVneo cells was 33% lower in the 1000 ng/mL BPS + EGF compared to the EGF group (*p* < 0.01) (Figure 5).

### 2.5. Tube Forming

**Figure 6:**
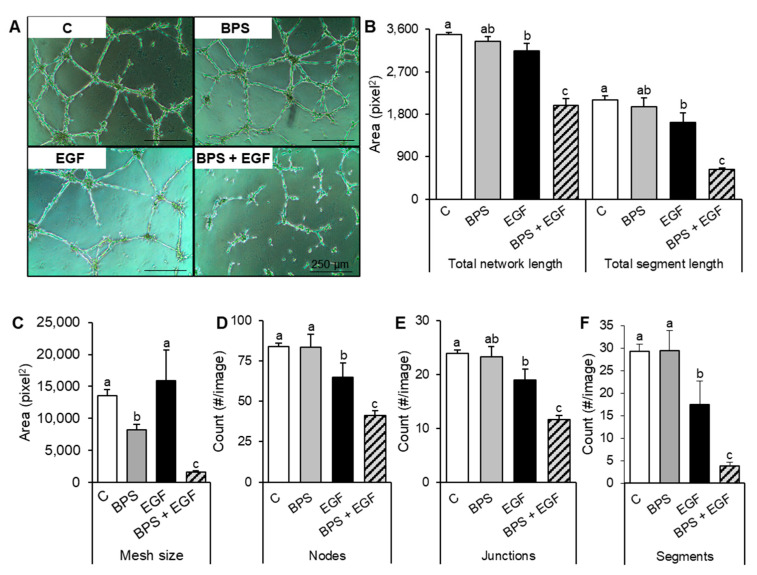
Effects of BPS on endovascular differentiation in trophoblast cells. (**A**) Representative images of HTR-8/SVneo cells were seeded in Matrigel and exposed for 18 h to the control (C; 0.1% DMSO), BPS (1000 ng/mL), EGF (30 ng/mL), or BPS (1000 ng/mL) + EGF (30 ng/mL) groups. (**B**–**F**) Quantification of tube formation parameters (mean ± SEM): (**B**) total network length and total segment length expressed as an area (pixel^2^), (**C**) mesh size expressed as an area (pixel^2^), (**D**) nodes expressed as the number of nodes per area, (**E**) junctions expressed as the number of junctions per area, (**F**) segments expressed as the number of segments per area. A generalized linear model was used compare treatments. Different letters denote statistical differences among treatment groups at *p* < 0.05 (*n* = 4 technical replicates/group).

To evaluate BPS’ ability to disrupt EVT endovascular differentiation, a tube forming assay was used. Evaluation of cell networks revealed that EGF exposed cells developed fewer meshes, nodes, and junctions compared to the control (*p* < 0.05), while BPS disrupted tube network formation by decreasing the mesh size (*p* = 0.04) of the tube network, but none of the other parameters studied. Co-exposure to BPS + EGF reduced the number and size of segments (tubes), meshes, junctions, and nodes when compared to either the control or the EGF group (*p* < 0.05) (Figure 6).

## 3. Discussion

In this study, we have demonstrated that BPS, an emerging bisphenol chemical, blocks EGF-mediated cell proliferation and invasion of first-trimester human EVT cells. In support of this, our data revealed that BPS (1) reduced EGFR phosphorylation and EGF internalization, (2) blocked EGF-induced cell proliferation and invasion, and (3) disrupted endovascular differentiation. The disruption of cell proliferation and invasion, critical processes for placental development, could result in placental defects [41]. Given that the placenta is among the tissues with the highest EGFR expression [42] and that BPS has previously reported toxic effects on primary isolated human cytotrophoblasts [24], there is justified need to evaluate BPS’ potential effect on placental outcomes in human studies.

Our current findings support that in human EVTs, BPS partially blocks EGF-mediated EGFR phosphorylation by competing with its natural ligand, EGF [24]. These results are in accordance with our previous findings in invasive breast adenocarcinoma cells and human term primary cytotrophoblasts [24,42]. Despite BPS’ observed effect on EGFR phosphorylation, this did not translate to phosphorylation of the downstream effectors, AKT and MAPK. The lack of measurable EGFR downstream phosphorylation was similar to that observed in breast cancer cells [24]. Longitudinal evaluation after EGFR activation should be used to capture the transient modulation of downstream effectors. We have also demonstrated that exposure to BPS lowered EGF internalization in EVT cells, reinforcing the premise that BPS acts as an EGFR antagonist and competes with EGF for EGFR binding. A similar inhibition of EGF internalization and EGFR phosphorylation has been reported for polychlorinated biphenyls (PCB-126 and PCB-153) and trans-nonachlor in a human epidermoid carcinoma cell line [39]. However, the direct effect of these chemicals on placental cell invasion and proliferation has not been demonstrated. How BPS interferes with EGF binding to EGFR, or whether BPS alters how EGFR is internalized remains to be evaluated. To note, reduced EGFR internalization can also reduce EGFR recycling back into the cell membrane [43]. Thus, reduced EGFR recycling may contribute to the dampened EGFR downstream signaling, compromising EGF-mediated processes in the placenta, such as trophoblast invasion and proliferation.

Given that placental cell proliferation is partly driven through an EGF-mediated mechanism, we tested whether BPS could affect the proliferative ability of EVT cells. Our results demonstrated that EGF, but not BPS, induced cell proliferation after 4 days in culture. When in a mixture (EGF + BPS at 1000 ng/mL), BPS blocked EGF-mediated proliferation in EVT cells. The time delay observed between BPS-mediated EGFR activation (at 15 min; Figure 1) and proliferation (Figure 3) is likely due to the time lag between EGFR activation and downstream activation of proliferation. While EGFR phosphorylation is elicited within minutes ([44]; Figure 1), the downstream effect leading to proliferation events is known to occur in hours to days [45,46]. This time lag has been reported when testing synthetic EGFR inhibitors in HER14 and K721A cells, with an effect on proliferation after 5 days of exposure [45]. BPS’ potential to affect cell proliferation is further supported by previous findings where low doses of BPS (0.1 to 1 nM) inhibited HTR-8/SVneo cell proliferation [47]. However, whether this inhibition of proliferation was due to BPS interference with EGFR activation remains unknown.

Critically important to placental function is the ability for EVTs to invade into the maternal decidua. Like cell proliferation, cell invasion can be stimulated by EGF [40], not just in cytotrophoblasts but also in carcinoma cells, such as breast adenocarcinoma MDA-MD-231 [48] and colon adenocarcinoma HCT-8/E11 cells [49]. Here, we have tested two in vitro models to evaluate BPS’ effect on cell invasion: (1) the classic Transwell invasion model that uses Matrigel as the extracellular matrix, and (2) an EVT invasion model that uses a 3D microfluidic chip to recreate the in vivo trophoblast microenvironment, incorporating continuous medium flow and shear stress within a fibronectin-based extracellular matrix [50]. Using the two models, we have demonstrated that BPS reduces EGF-mediated invasion in HTR-8/SVneo cells in a dose-dependent manner, with 100 ng/mL BPS attenuating and 1000 ng/mL BPS fully blocking EGF-induced cell invasion. The fact that BPS attenuated EGF-mediated invasion at an environmentally relevant dose has important implications in human pregnancies because defects of EVT invasiveness can result in deficient spiral artery remodeling. This, in turn, is associated with abnormal placentation and pregnancy outcomes, such as the development of preeclampsia [51,52], fetal growth restriction [53], and early pregnancy loss [54]. Commonly used in the manufacturing of epoxy glues, food can coatings, thermal receipt papers, textile dyes, and tanning agents [55], over 80% of the population is ubiquitously exposed to BPS [6]. In this study, the lowest BPS dose that exerted a significative effect on cell invasion was 100 ng/mL BPS, a dose within the urinary BPS concentration range observed in the U.S. (0.07 to 211.9 ng/mL; [10]). While in vivo exposure to BPS can result in cell fusion defects in an ovine model [23], effects of BPS in human pregnancies have been associated with both higher [56] and lower [57] birth weight and birth length. Thus, whether BPS can interfere with EGF signaling, in vivo, in humans, resulting in poor pregnancy outcomes remains to be determined.

To test whether BPS could simultaneously interfere with trophoblast proliferation and invasion, we used a tube-like formation assay [58], and observed that BPS disrupted tube network formation by decreasing the mesh size of the tube network, but none of the other parameters studied. This finding supports a previous study that reported how low doses of BPS (1 nM) inhibit tube formation in HTR-8/SVneo cells in association with lower expression of genes responsible for angiogenesis such as VEGF, PCNA, and ICAM1 [47]. In support of our previous findings on proliferation and invasion (Figure 3, Figure 4 and Figure 5), BPS in the presence of EGF, almost fully inhibited tube formation. Given the strength of this effect compared to other endpoints tested (i.e., proliferation, invasion, and endocytosis), it is possible that BPS elicited this effect via EGFR inhibition, or other pathways that control gap junction intercellular communication [59], or that intersect with angiogenic regulation [60]. For instance, cell-to-cell communication that involves Notch signaling is crucial for the formation of complex multicellular structures such as blood vessel networks [60]. Since the estrogen receptor and EGFR pathways can regulate Notch signaling in opposite directions [61], the effect the BPS and EGF mixture has on inhibition of tube formation may stem from an inhibition of the EGFR pathway associated with an induction of the estrogen receptor pathway [62]. However, whether in vivo exposure to BPS alters angiogenesis during placental development remains to be determined.

In conclusion, we have demonstrated that BPS can block EGF internalization and EGFR phosphorylation in human extravillous trophoblast cells. Importantly, this effect resulted in a BPS-induced reduction in EGF-mediated extravillous trophoblast cell invasion and proliferation. Of translational relevance is the fact that the invasion effect was achieved at a dose within the range of BPS observed in human urine. Given the importance of EGFR in placental development, including cytotrophoblast proliferation, differentiation, and invasion, these and previous findings [23,24] suggest that gestational exposure to BPS may result in placental dysfunction. Other tissues with high EGFR expression such as breast, skin, and liver may also be targets of BPS-mediated EGFR dysregulation.

## 4. Materials and Methods

### 4.1. Exposure Chemicals

Chemicals used in this study were: bisphenol S (4,4′-sulfonyldiphenol, Cat#: 80-09-11, Acros Organics, Geel, Belgium), human EGF (Cat#: E9644, Sigma Aldrich, St. Louis, MO, USA), Alexa Fluor 647-conjugated EGF (biotinylated EGF complexed to Alexa Fluor 647 Streptavidin, Cat#: E13345, Thermo-Scientific, Philadelphia, PA, USA), and dimethyl sulfoxide (DMSO, Cat#: BP231-100, Thermo-Fisher, Rockford, IL, USA). DMSO was used as the vehicle and added to a final concentration of 0.1% in all groups.

### 4.2. HTR-8/SVneo Cell Culture

To test BPS’ EGFR antagonism, HTR-8/SVneo cells were used. HTR-8/SVneo is a first-trimester human extravillous trophoblast cell line with EGFR expression [63] that proliferates and invade in response to EGF [49]. HTR-8/SVneo were maintained in basic medium, Dulbecco’s modified Eagle’s medium/F12 medium (Cat#: 124000-024, MilliporeSigma, Saint Louis, MO, USA) supplemented with 10% of fetal bovine serum (FBS), 2 mM L-glutamine, 10 mM HEPES, 100 IU/mL penicillin, and 100 μg/mL streptomycin. Cells were cultured in 5% CO2 at 37 °C.

### 4.3. Cell Proliferation Assay

To determine whether BPS could inhibit the EGF-induced HTR-8/SVneo cell proliferation, the CellTiter-glo luminescent cell viability assay kit (Cat#: G8091, Promega, Madison, WI, USA) was used per manufacturer’s instructions. ATP levels correlate linearly with the number of viable cells and growth kinetics in human cancer cell line cultures [64,65]. Cells were seeded into clear 96-well plates at 2.5 × 10^3^ cells per well and exposed to four different culture conditions: control (vehicle: 0.1% DMSO), BPS (1000 ng/mL), EGF (30 ng/mL), or BPS + EGF for 2, 3, 4, 5, or 6 days. All treatments were performed in triplicate. After exposure, cellular proliferation was measured by intracellular ATP levels in metabolically active cells. Cells were incubated with 100 µL of CellTiter-Glo per well for 10 min and luminescence was recorded using a multi-mode microplate reader (SpectraMax M5e, Molecular Devices LLC, Sunnyvale, CA, USA). Results were expressed as relative light units (RLU).

### 4.4. Western Blotting

To test whether BPS could affect EGF-mediated protein abundance and phosphorylation, HTR-8/SVneo cells were cultured in growth medium in a 6-well plate and exposed in triplicate to four different culture conditions: control (vehicle: 0.1% DMSO), BPS (1000 ng/mL), EGF (30 ng/mL), or BPS (1000 ng/mL) + EGF (30 ng/mL). After 15 min, cells were harvested for protein quantification as previously described [24]. Protein extraction was performed using RIPA lysis buffer (Cat#: N653, VWR Life Science, San Francisco, CA, USA) containing 20% 1 M NaF, 1 mM Na_3_VO_4_ and 1% protease inhibitor cocktail (Cat#: M221, VWR Life Science, San Francisco, CA, USA). Protein concentration was determined using a Pierce BCA protein assay kit (Cat#: 23225, Thermo-Fisher, Rockford, IL, USA). Twenty micrograms of protein per sample from cell lysates were subjected to electrophoresis on a 10% SDS-polyacrylamide gel (120 V for 60 min). Protein was then transferred from the gel to a nitrocellulose membrane (200 mA for 90 min) and subjected to Western blotting. In brief, membranes were blocked with 5% nonfat dry milk in tris-buffered saline (TBS) containing 0.03% tween-20 (block solution) and incubated with primary antibodies diluted in block solution overnight at 4 °C. Primary antibodies used were: anti-EGFR, anti-ERK, anti-β-actin, anti-AKT, anti-phospho-Akt (Tyr 204), anti-phospho-EGFR (Tyr 1068) and anti-phospho-p44/42 MAPK (Thr202/Tyr204) (Supplementary Table S1). After three washes with TBS with 0.03% Tween-20 detergent (TBS-T), membranes were incubated with secondary antibodies goat anti-mouse HRP-conjugated, and goat anti-rabbit HRP-conjugated diluted 1:5000 in block solution for 1 h at room temperature in the dark. Western Bright ECL (Cat#: K12045, Advansta, Menlo Park, CA, USA) was used for enhanced chemiluminescence and visualized on a Thermo-Scientific MYECL Imager (Cat#: K12045, Thermo Scientific, Waltham, MA, USA). Quantification of band intensities was performed using ImageJ software [66]. Differences in protein loading were accounted for by normalizing the target protein band by the control β-actin band for each sample.

### 4.5. EGF Endocytosis Assay

EGF internalization was evaluated in HTR-8/SVneo cells using a modified EGF endocytosis assay [24]. After overnight serum starvation, cells were pre-treated with BPS (1 or 10 μg/mL) in 0.1% DMSO for 5 min, followed by a 5 min co-exposure with BPS (1 and 10 µg/mL) and Alexa Fluor Texas Red-conjugated EGF (100 ng/mL; biotinylated EGF complexed to Alexa Fluor 647 Streptavidin, Cat#: E13345, Thermo-Scientific, Philadelphia, PA, USA). The positive control group was exposed to 0.1% DMSO. Negative controls were treated with 100 ng/mL unlabeled recombinant human EGF (Cat#: E9644, I3390, MilliporeSigma, Saint Louis, MO, USA). Cells were then washed with pre-warmed PBS and incubated at 37 °C in serum-free IMD medium for 60 min, then fixed using a 1:1 methanol:acetone solution at −20 °C for 20 min, followed by three TBS washes. Cell nuclei were stained with DAPI (1:1000). For EGF endocytosis quantification, three random images (40X magnification) per well for a total of 3 wells per treatment group were obtained using (Lionheart FX, BioTek Instruments Inc, Winooski, VT, USA). Filter sets for 350 and 590 nm were used for detection of DAPI and Alexa Fluor Texas Red-conjugated EGF, respectively. Over 1800 cells from randomized fields from each group were analyzed using the software CellProfiler [67]. Total labeled EGF was normalized to the total number of nuclei.

### 4.6. Transwell Cell Invasion

To test whether BPS could affect EGF-mediated cell invasion, Matrigel pre-coated Transwell cell culture inserts (24-wells, 8 µm pore size; Corning, Tewksbury, MA, USA) were placed in a 24-well plate and rehydrated at room temperature for 2 h, as previously described [50]. HTR-8/SVneo cells were then trypsin harvested and seeded at 50,000 cells per insert in 250 µL serum free medium. Cells were allocated to one of four groups: control (vehicle: 0.1% DMSO), EGF (30 ng/mL), BPS (1000 ng/mL), or the combination of EGF (30 ng/mL) + BPS at either 10, 100, or 1000 ng/mL. Thereafter, HTR-8/SVneo cell’s basic medium was supplemented with 10% FBS, and the exposure chemicals mentioned above were added to the lower insert chamber. After a 16 h incubation, non-invading cells were removed by using a cotton swab on the upper side of the membrane. The invaded cells that penetrated the membrane were fixed with 10% neutral-buffered formalin for 30 min at room temperature, then stained with DAPI for nuclear quantification. For quantification of number of cells, four random images per well for a total of 4 wells per treatment group were obtained using an Olympus BX41 fluorescence microscope and Olympus DP71 camera. A 350 nm filter was used for detection of DAPI. Cells (mean ± SD: 441 ± 109 per group) were then analyzed from randomized fields for each group.

### 4.7. 3D Microfluidic Chip Cell Invasion

To test cell invasion in a platform that better resembles the placental microenvironment, we used a 3D polydimethylsiloxane (PDMS) microfluidic chip as previously described [50]. This platform reproduces key elements of the placental microenvironment, including the extracellular matrix (ECM), and incorporates dynamic medium flow while allowing for real-time monitoring, imaging, and evaluation of trophoblast cell invasion. HTR-8/SVneo cells (GFP tagged as previously described [50]) were seeded in the outer chamber, which was pre-coated with fibronectin. The central compartment was flushed with cell medium supplemented with 10% FBS. The treatment groups were: control (DMSO, 0.1% *v*/*v*), EGF (30 ng/mL), and EGF + BPS (1000 ng/mL). The outer channels were flushed with low serum (1% FBS) cell medium containing DMSO (0.1% *v*/*v*; control), EGF (30 ng/mL), or EGF + BPS (1000 ng/mL). The number of HTR-8/SVneo cells that invaded into the central compartment were quantified 72 h post-exposure.

### 4.8. Endovascular Differentiation Assay

To test whether BPS could affect endovascular differentiation of trophoblast cells, HTR-8/SVneo cells were seeded at low density (40,000 cells per 60 mm plate) and kept in basic medium. Matrigel (Cat#: 356234, Corning, Woodland, CA, USA) kept at 4 °C was used to coat a 24-well plate and allowed to polymerize at 37 °C for 30 min. Cell were trypsin harvested and the resultant pellet was diluted in basic medium with one of the following treatment groups: control (vehicle: 0.1% DMSO), BPS (1000 ng/mL), EGF (30 ng/mL), or BPS (1000 ng/mL) + EGF (30 ng/mL). Finally, 40,000 HTR-8/SVneo cells/well suspended in 400 µL of basic medium were added and incubated overnight at 37 °C and 5% of CO_2_. After an 18 h incubation, formation of cell networks was examined visually on a Zeiss IM light microscope equipped with an Olympus DP71 camera. This assay was completed in triplicate.

### 4.9. Statistical Analysis

Quantitative data comprising invasive cells, protein abundance, fluorescent signal and tube-like formation parameters were compared between groups by using a generalized linear model (MIXED procedure) that allows adjustment of means while considering an effect from exposure time. The model used included treatment groups and time as fixed effects. Data were tested for normality by the Anderson–Darling normality test. LSMEANS was used to adjust the means and to compare treatments. Significance was set at *p* < 0.05. All data were analyzed using SAS version 9.4.

## Supplementary Materials

Supplementary materials can be found at https://www.mdpi.com/article/10.3390/ijms23020671/s1.
