# Supplementary material for: Bisphenol S Impairs Invasion and Proliferation of Extravillous Trophoblasts Cells by Interfering with Epidermal Growth Factor Receptor Signaling
Source: Int J Mol Sci. 2022 Jan 8;23(2):671. doi: 10.3390/ijms23020671 (PMC8776214; doi:10.3390/ijms23020671)
Supplement: Supplementary file 1 [file ijms-23-00671-s001.zip › ijms-1474200-supplementary.pptx]

## Slide 1
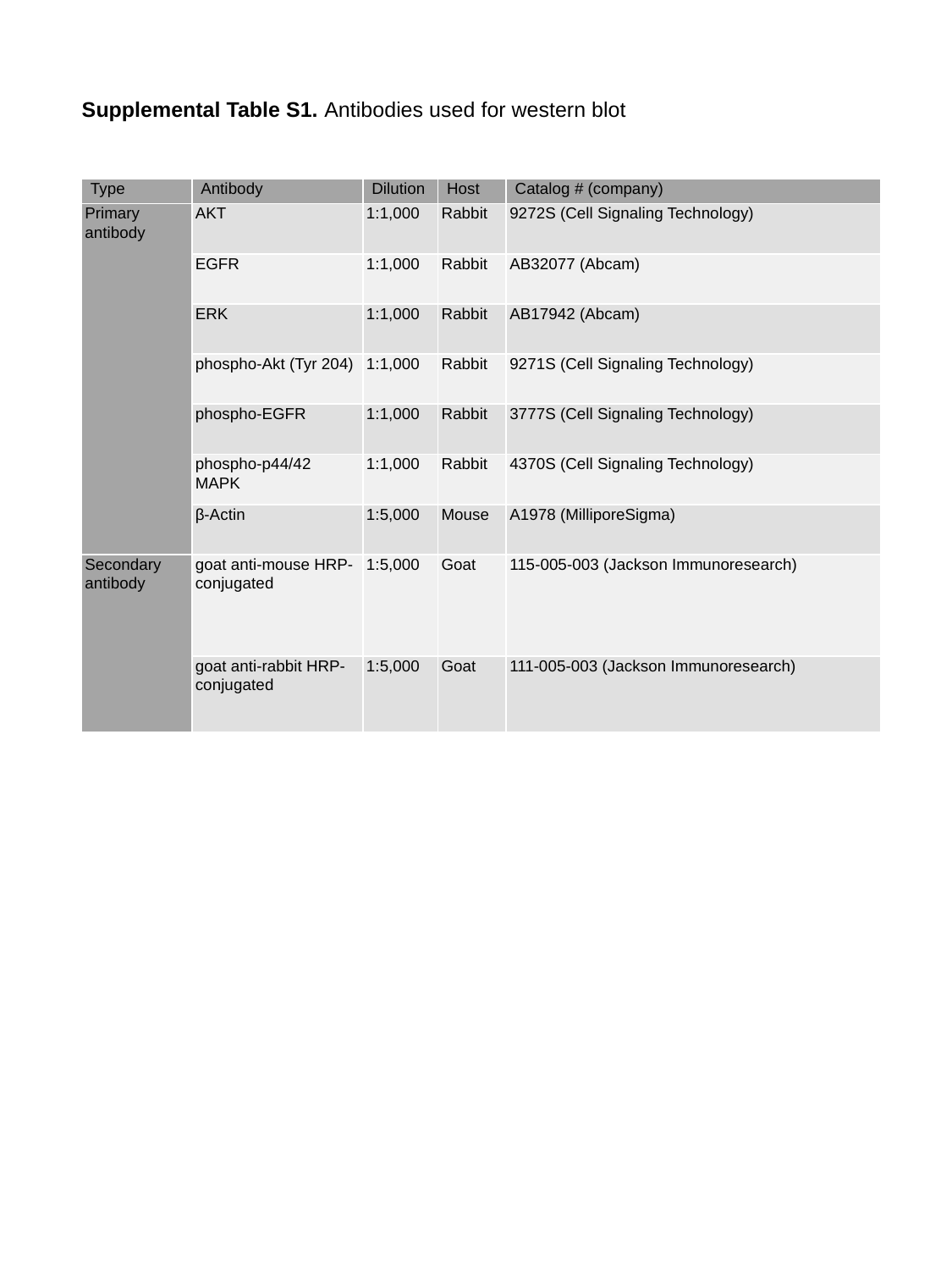

Supplemental Table S1. Antibodies used for western blot
IF: immunofluorescence, WB: western blotting
| Type | Antibody | Dilution | Host | Catalog # (company) |
| --- | --- | --- | --- | --- |
| Primary antibody | AKT | 1:1,000 | Rabbit | 9272S (Cell Signaling Technology) |
| | EGFR | 1:1,000 | Rabbit | AB32077 (Abcam) |
| | ERK | 1:1,000 | Rabbit | AB17942 (Abcam) |
| | phospho-Akt (Tyr 204) | 1:1,000 | Rabbit | 9271S (Cell Signaling Technology) |
| | phospho-EGFR | 1:1,000 | Rabbit | 3777S (Cell Signaling Technology) |
| | phospho-p44/42 MAPK | 1:1,000 | Rabbit | 4370S (Cell Signaling Technology) |
| | β-Actin | 1:5,000 | Mouse | A1978 (MilliporeSigma) |
| Secondary antibody | goat anti-mouse HRP-conjugated | 1:5,000 | Goat | 115-005-003 (Jackson Immunoresearch) |
| | goat anti-rabbit HRP-conjugated | 1:5,000 | Goat | 111-005-003 (Jackson Immunoresearch) |
